# Return to work following laparoscopic‐assisted resection or open resection for rectal cancer: Findings from AlaCaRT—Australasian Laparoscopic Cancer of the Rectum Trial

**DOI:** 10.1002/cam4.3623

**Published:** 2020-12-06

**Authors:** Chi Kin Law, Kate Brewer, Chris Brown, Kate Wilson, Lisa Bailey, Wendy Hague, John R. Simes, Andrew Stevenson, Michael Solomon, Rachael L. Morton, the Australasian Gastro‐Intestinal Trials Group (AGITG) ALaCaRT investigators

**Affiliations:** ^1^ NHMRC Clinical Trials Centre The University of Sydney Camperdown NSW Australia; ^2^ Faculty of Medicine and Biomedical Sciences University of Queensland Herston Qld Australia; ^3^ Institute of Academic Surgery Royal Prince Alfred Hospital University of Sydney Sydney NSW Australia

**Keywords:** clinical trial, income, laparoscopy, open abdomen techniques, rectal neoplasms, return to work, socioeconomic factors

## Abstract

**Background:**

Maintaining employment for adults with cancer is important, however, little is known about the impact of surgery for rectal cancer on an individual's capacity to return to work (RTW). This study aimed to determine the impact of laparoscopic vs. open resection on RTW at 12 months.

**Methods:**

Analyses were undertaken among participants randomized in the Australian Laparoscopic Cancer of the Rectum Trial (ALaCaRT), with work status available at baseline (presurgery), and 12 months. Multivariable logistic regression, adjusted for sociodemographic and clinical characteristics estimated the effect of surgery on RTW in any capacity, or return to preoperative work status at 12 months.

**Results:**

About 228 of 449 (51%) surviving trial participants at 12 months completed work status questionnaires; mean age was 62 years, 66% males, 117 of these received laparoscopic resection (51%). Of 228, 120 were employed at baseline (90 full‐time, 30 part‐time). Overall RTW in 120 participants in paid work at baseline was 78% (84% laparoscopic, 70% open surgery). Those employed full‐time were more likely to RTW at 12 months (OR, 3.55; 95% CI, 1.02–12.31). Those with distant metastases at baseline were less likely to RTW (OR, 0.07; 95% CI, <0.01–0.83). Laparoscopic surgery was associated with a higher rate of RTW but did not reach statistical significance (OR 2.88; 95% CI, 0.95–8.76).

**Conclusions:**

Full‐time work presurgery and the presence of metastatic disease predicts RTW status at 12 months. A laparoscopic‐assisted surgical approach to rectal cancer may facilitate more patients to RTW, however, larger sample sizes are likely needed to confirm this result.

## Graphical abstract

Rectal cancer patients with laparoscopic‐assisted surgery have a slightly but not significantly higher return to work rate at 12 months than those with open rectal resection. Results of this randomised controlled trial highlight the importance of discussing employment disruption and retention strategies with adults undergoing surgery for rectal cancer

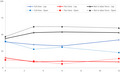

## 1 INTRODUCTION

Rectal cancer has become an emerging public health issue among working‐age adults in Australia and New Zealand due to an increased prevalence of obesity, alcohol consumption and dietary intake of red and processed meats.^1^, ^2^ According to the Australian Institute of Health and Welfare (AIHW) 2015 data, the incidence rate of rectal cancer for ages 30–64 years was 19.3 per 100,000 and the corresponding mortality rate was 6.5.^3^ Employed individuals with rectal cancer face many challenges to their work life as treatments are often disruptive to their employment, earnings, and other role activities.^4^, ^5^, ^6^ One study among employed middle‐aged people with colorectal cancer, reported 27% stopped working and 19% reduced their work hours at 1‐year postdiagnosis.^5^ Stopping or reducing work may be a source of financial and psychological stress for cancer survivors,^6^, ^7^, ^8^, ^9^ which adversely affects their health‐related quality of life (HRQoL) and economic security.^4^, ^5^, ^8^

Laparoscopic‐assisted resection has been widely used in colon but not rectal cancer surgery, since it was first described in 1992,^10^ with reported benefits of less intraoperative blood loss, fewer adhesions, shorter hospital stay, and faster return to work (RTW).^11^, ^12^, ^13^, ^14^, ^15^, ^16^, ^17^ In achieving better short‐term outcomes, proponents of the laparoscopic approach have persistently advocated this technique for rectal cancer treatment.^18^

Using an open laparotomy approach, resection of the rectum often involves a more complicated and morbid procedure than for other gastro‐intestinal cancers and more precise dissection to reduce the chance of cancer recurrence.^18^ However, the noninferiority of the laparoscopic approach to conventional open resection for rectal cancer treatment based on pathological outcomes was not established in the Australian Laparoscopic Cancer of the Rectum Trial (ALaCaRT)^19^, ^20^ or the American College of Surgeons Oncology Group Z6051 trial,^21^ even though 2 year oncologic outcomes were not significantly different.^20^ In addition to clinical outcomes, the effect of laparoscopic‐assisted surgery on RTW for rectal cancer survivors has never been evaluated in a randomized trial.

The purpose of this analysis was to examine the effect of laparoscopic compared with open resection for rectal cancer on RTW among rectal cancer survivors at 12 months participating in ALaCaRT, after adjusting for sociodemographic and clinical characteristics.

## 2 METHODS

### 2.1 Study participants

Data from the first 12 months after enrolment and surgery for ALaCaRT were used in this prospective analysis. ALaCaRT was a multicenter randomized, noninferiority, phase 3 trial evaluating the safety, and efficacy of laparoscopic resection versus open surgery for rectal cancer.^19^, ^20^ ALaCaRT participants were adults aged 18 years or older, with a histological diagnosis of adenocarcinoma of the rectum within 15 cm of the anal verge, and a life expectancy of at least 12 weeks.^19^ Four hundred and seventy‐five patients from 24 hospitals in Australia and New Zealand were recruited between March 2010 and November 2014, with 473 eligible for analysis.^19^ All ALaCaRT participants were randomized 1:1 to undergo laparoscopic or open surgery stratified by site of the tumor, the registering surgeon, the planned operative procedure (low anterior resection or abdominoperineal resection), body mass index (BMI), preoperative radiotherapy and distant metastases. Central ethics approval was obtained by the Sydney Local Health District human research ethics committee. Individual sites not covered by the central approval obtained local approval. All participants gave written informed consent before trial randomization. The study protocol has been reported in detail previously.^19^

ALaCaRT participants were included in the current analysis if they reported their work status in the “Labour force and Income impacts of illness survey” (the survey) at study baseline prior to surgery and 12 months after surgery. The survey collected participants' socioeconomic data (including family composition, education level, work status, and annual income) at study baseline prior to surgery, 3, 6, and 12 months after surgery. Clinical data and surgical outcomes of participants were extracted from the trial database.

### 2.2 Study variables

**FIGURE 1:**
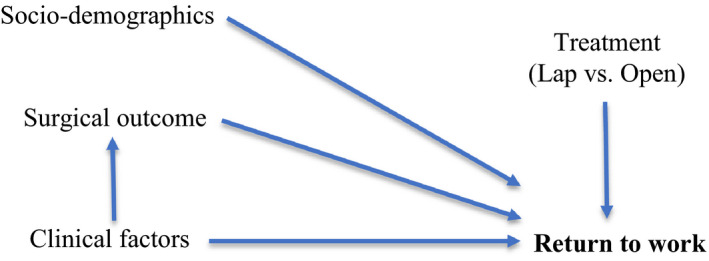
Causal diagram assessing the relationships between sociodemographic and clinical characteristics, treatment type and surgical outcomes on return to work for rectal cancer survivors

The main outcome for this analysis was the RTW rate at 12 months following either type of surgery. Figure 1 depicts a causal diagram (i.e. logic model) ^22^, ^23^ of the possible associations with sociodemographic, clinical characteristics, and surgical outcomes on RTW at 12 months. To define “RTW”, two measures were calculated: (a) participants in paid full‐time or part‐time paid employment at 12 months (yes, no); (b) return to preoperative work status or full‐time work at 12 months (yes, no). Participants who were unemployed or not in the labor force at study baseline were excluded from these analyses.

Explanatory variables included type of surgery (laparoscopic‐assisted vs. open resection), surgical/pathology composite outcome (successful vs. unsuccessful) and participants' sociodemographic and clinical characteristics at baseline. We used the pathological criterion for successful resection, which comprises complete total mesorectal excision (TME), clear circumferential margins (≥1 mm) and clear distal margins (≥1 mm), as described previously.^19^, ^20^ Clinical factors included BMI kg/m^2^ (<25, 25–30, ≥30); site of tumor (high: 10–15 cm from the anal verge, middle: 5–10 cm, low:<5 cm); tumor stage (T1, T2, T3); nodal status (N0, N1, N2); distant metastases (M0, M1); planned operative procedure (low anterior resection, abdominoperineal resection); preoperative radiotherapy (yes, no); performance status measured by Eastern Cooperative Oncology Group Scale (0–4).^24^ Socioeconomic factors included age in years at randomization; sex (male, female); family composition (couple with dependent children, single parent, couple only, living alone); work status at baseline (full‐time paid work, part‐time paid work, unemployed looking for work, not in labor force [i.e. do not have a job and not looking for work]); highest educational attainment (university degree, certificate/Diploma, high school, or leaving certificate, year 9 or below/never attended school); usual yearly personal income before tax (<AU$29,999; $30,000–$69,999; $70,000–$149,999; $150,000 or more).

### 2.3 Statistical methods

Descriptive statistics are presented for participants' sociodemographic and clinical characteristics at baseline, type of surgery (randomization allocation as per intention to treat principle), and surgical outcome. Variables hypothesized to be associated with RTW, were identified a priori and are presented in Figure 1. Univariate analyses included the Pearson chi‐square (χ^2^) tests for categorical variables and the Student's *t*‐tests for continuous variables. Multivariate logistic regression models were built using known associated variables and backward elimination methods to estimate the effect of treatment, surgical outcome, and other factors associated with RTW at 12 months after the index procedure (surgery); with adjustment for age, sex, and cancer TNM stage.^25^, ^26^, ^27^, ^28^, ^29^, ^30^ Odds ratios (ORs) with conventional 95% CIs were presented and all quoted p‐values referred to the comparison with the specified referent category, with *p* < 0.05 for statistical significance. All analyses were conducted in SAS 9.4, Windows version (SAS Institute).

### 2.4 Sensitivity analyses

To ascertain factors associated with a return to preoperative work status, we performed separate logistic regressions among the participants who had returned to paid work at 12 months, excluding those who did not return to their preoperative work status (sensitivity analysis 1, [SA1]). To investigate the impact of missing data on the sample who returned to work at 12 months, and who completed the work status questionnaire at 3‐ or 6‐month time points (*n* = 102), we conducted a separate analysis (SA2). To explore whether the inclusion of participants who had died during the 12‐month follow‐up period influenced the impact of type of surgery on RTW, a separate sensitivity analysis was conducted including those who completed the baseline measure (SA3). The 12‐month RTW and return to preoperative work status measure was coded as “No” for deceased participants.

## 3 RESULTS

**FIGURE 2:**
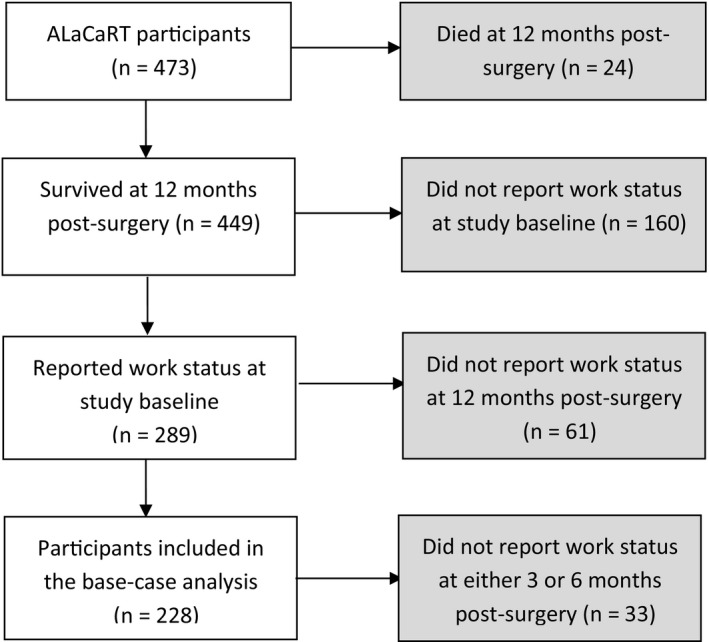
ALaCaRT participants included in the return to work analysis. ALaCaRT, Australian Laparoscopic Cancer of the Rectum Trial

Of 473 ALaCaRT participants, 449 survived ≥12 months and 228 of these (51%) completed the work status questionnaire at baseline and 12 months. (Figure 2) To assess the representativeness of this sample, comparisons were conducted between ALaCaRT participants who completed the work status questionnaire and those did not. Respondents in our analysis were slightly younger, had tumor in the middle part of rectum rather than the low part of rectum, were less likely to have tumor‐stage 3 (T3) cancer, and receive preoperative radiotherapy compared with nonrespondents.(Table 1) Baseline characteristics of the study sample by treatment arm (laparoscopic‐assisted surgery *n* = 117; open surgery *n* = 111) are shown in Table 2. No significant difference in baseline characteristics by surgery type was identified.

**TABLE 1:** Baseline characteristics and surgical outcomes among ALaCaRT participants by whether reported their work status in the labor force and income impacts of illness survey (the survey) at baseline and 12‐month after surgery.

Variable	Description (%, unless specified)	Included in RTW analysis		All (n = 473)	*p*‐value
		Yes (n = 228)	No (n = 245)		
Sex	Males	65.8	65.7	65.8	0.986
	Females	34.2	34.3	34.2	
Age	Mean age (years)	61.7	64.9	63.4	**0.01**
Age group	Below 45	8.3	6.5	7.4	**0.004**
	45–54	16.2	15.1	15.6	
	55–64	33.8	22.5	27.9	
	65–75	28.5	30.6	29.6	
	75 or above	13.2	25.3	19.5	
Treatment arm	Laparoscopic‐assisted surgery	51.3	49.4	50.3	0.68
Surgical outcome	Successful resection	86.8	83.3	85.0	0.28
Preoperative radiotherapy		43.4	55.1	49.5	**0.01**
BMI	Below 25	31.1	36.3	33.8	0.33
	25–30	43.0	42.9	42.9	
	30 or above	25.9	20.8	23.3	
Site of tumor	High	23.3	20.4	21.8	**0.01**
	Middle	49.1	38.0	43.3	
	Low	27.6	41.6	34.9	
T‐stage	T1	5.3	7.4	6.4	**0.04**
	T2	33.9	23.8	28.7	
	T3	60.8	68.8	64.9	
N‐stage	N0	51.5	47.4	49.4	0.52
	N1	35.2	37.1	36.2	
	N2	12.8	15.5	14.2	
	Nx	0.4	0	0.2	
M‐stage	M1	5.3	4.5	4.9	0.70
Performance status (ECOG)	0	82.4	78.3	80.3	0.23
	1	16.7	18.8	17.8	
	2	0.9	2.9	1.9	
Planned operative procedure	Abdominoperineal resection	7.0	7.8	7.4	0.76

Abbreviation: ALaCaRT, Australian Laparoscopic Cancer of the Rectum Trial; ECOG, Eastern Cooperative Oncology Group Scale.

**TABLE 2:** Baseline characteristics of 228 ALaCaRT participants who completed the work status questionnaire at baseline and 12‐months after surgery, by treatment arm.

Variable	Description (%, unless specified)	Treatment arm		p‐value
		Laparoscopic‐assisted (n = 117)	Open (n = 111)	
Sex	Males	68.4	63.1	0.40
	Females	31.6	36.9	
Age	Mean age (years)	61.7	61.7	0.99
Age group (years)	Below 45	7.7	9.0	0.20
	45–54	17.1	15.3	
	55–64	36.8	30.6	
	65–74	22.2	35.1	
	75 or above	16.2	9.9	
Surgical outcome	Successful resection	82.9	91.0	0.07
Preoperative radiotherapy	Yes	47.0	39.6	0.26
BMI	Below 25	30.8	31.5	0.98
	25–30	43.6	42.3	
	30 or above	25.6	26.1	
Site of tumor	High	24.8	21.6	0.83
	Middle	48.7	49.6	
	Low	26.5	28.8	
T‐stage	T1	7.7	2.7	0.24
	T2	32.5	35.5	
	T3	59.8	61.8	
N‐stage	N0	43.9	59.5	0.08
	N1	38.8	31.5	
	N2	16.4	9.0	
	Nx	0.9	0	
M‐stage	M1	6.8	3.6	0.27
Performance status	0	78.6	86.5	0.16
	1	19.7	13.5	
	2	1.7	0	
Planned operative procedure	Abdominoperineal resection	8.6	5.4	0.35
Family composition	Couple with dependent children	18.8	23.6	0.63
	One person with dependent children	1.7	1.8	
	Couple only	53.9	55.5	
	Living alone	25.6	19.1	
Education level	University degree	28.5	19.3	0.45
	Certificate/ diploma	25.0	29.4	
	High school	33.6	37.6	
	Year 9 or below	12.9	13.8	
Usual yearly personal income before tax (AU$)	Less than $30,000	43.6	48.7	0.21
	$30,000–$69,999	30.8	32.4	
	$70,000–$149,999	16.2	16.2	
	More than $150,000	9.4	2.7	
Main source of income	Wage/salaries	35.3	40.0	0.14
	Self‐employed	17.2	11.8	
	Government benefits	25.9	30.9	
	Farm	2.6	0	
	Other^a^	13.8	16.4	
	No source	5.2	0.9	
Work status	Full‐time paid work	39.3	39.6	0.89
	Part‐time paid work	14.5	11.7	
	Unemployed	2.6	1.8	
	Not in labor force	43.6	46.9	
Main reason for not working	Own ill health—cancer related	16.7	12.7	0.40
	Own ill health—other illness	9.3	5.5	
	Care‐giving for sick/disabled person	1.9	5.5	
	Retired	68.5	76.4	
	Others^b^	3.7	0	

Abbreviation: ALaCaRT, Australian Laparoscopic Cancer of the Rectum Trial.

^a^ Other sources of income included the Age Pension, Veterans’ pension, superannuation, rental income, and share dividends.

^b^ Other reasons included redundancy and work‐based issues.

### 3.1 Change in work status distribution

**FIGURE 3:**
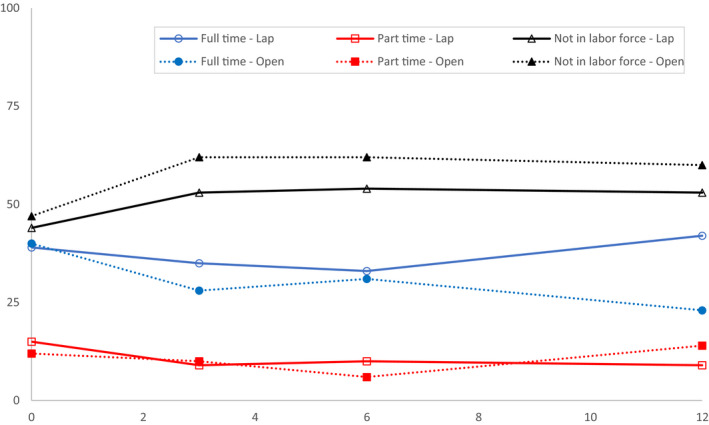
Proportion of ALaCaRT participants by treatment arm, employed full‐time, part‐time or not in the labor force at baseline, 3, 6 and 12‐months (n = 228). Number and proportion of ALaCaRT participants by treatment arm, employed full‐time, part‐time or not in the labor force at baseline, 3, 6 and 12‐months. ALaCaRT, Australian Laparoscopic Cancer of the Rectum Trial

The distribution of work status between the two treatment arms across the study assessments is shown in Figure 3. For rectal cancer survivors in the laparoscopic‐assisted surgery group, the proportion not in the labor force increased from 44% at baseline to 53% (i.e. 9% dropped out of the workforce) 12 months after surgery. This change was mainly driven by a reduction in the proportion of participants in part‐time paid work from 15% to 9% over the same period. The proportion of people receiving open resection and not in the labor force at 12 months increased from 47% at baseline to 60% (13% dropped out of the workforce). This change was mostly due to a reduction in the proportion of participants in full‐time paid work from 40% to 23% over the same period.

### 3.2 Return to work

Table 3 illustrates that work status at baseline was a strong predictor of participants' RTW at 12 months. A high proportion (82%) of participants in full‐time paid work at baseline returned to work 12 months after surgery. Sixty‐three percent of participants in part‐time paid work at baseline returned to work over the same period. Two participants (2%) who were not working at baseline (either unemployed or not in the labor force) entered the workforce 12 months after surgery.

**TABLE 3:** Change in work status from baseline to 12 months.

Work status at baseline	Work status at 12 months		
	Full‐time paid work (n = 68)	Part‐time paid work (n = 27)	Unemployed/not in labor force (n = 133)
Full‐time paid work (n = 90)	63 (70.0%)	11 (12.0%)	16 (18.0%)
Part‐time paid work (n = 30)	5 (16.7%)	14 (46.7%)	11 (36.7%)
Unemployed/ not in labor force (n = 108)	0 (0%)	2 (1.9%)	106 (98.1%)

Results of the logistic regression analysis for RTW and return to preoperative work status at 12 months are shown in Table 4. The unadjusted OR of RTW at 12 months for participants receiving laparoscopic resection was 2.25 (95% CI, 0.93–5.44). After adjustment for relevant sociodemographic and clinical factors, the effect of laparoscopic‐assisted surgery on RTW was higher but not statistically significant (OR, 2.88; 95% CI, 0.95–8.76). Multivariate analyses showed participants in full‐time paid work at baseline were more likely to RTW at 12 months (OR, 3.55; 95% CI, 1.02–12.31), while those with distant metastases (OR, 0.07; 95% CI, 0.01–0.83) were less likely to RTW during the 12‐month follow‐up period.

**TABLE 4:** Multivariable analysis of return to work and return to preoperative work status for ALaCaRT participants at 12 months.

Variable		Return to work at 12 months (n = 120)		Return to preoperative work status or full‐time work at 12 months (n = 120)	
		OR	95% CI	OR	95% CI
Treatment arm	Open	1	Reference	1	Reference
	Laparoscopic assisted	2.88	0.95–8.76	2.01	0.83–4.86
Surgical outcome	Unsuccessful resection	1	Reference	1	Reference
	Successful resection	3.81	0.81–17.84	2.00	0.57–7.00
Sex	Females	1	Reference	1	Reference
	Males	1.20	0.35–4.15	1.81	0.73–4.51
Age (years) at randomization		0.95	0.90–1.01	0.98	0.95–1.03
Tumor stage	T1	1	Reference	1	Reference
	T2	2.21	0.31–15.63	0.83	0.14–4.94
	T3	1.21	0.17–8.45	0.39	0.06–2.34
Nodal status	N0	1	Reference	1	Reference
	N1	0.61	0.16–2.29	1.64	0.54–4.96
	N2	10.42	0.64–170.05	6.03	0.96–37.88
Distant metastasis	M0	1	Reference	1	Reference
	M1	**0.07**	**0.01–0.83**	0.38	0.05–2.97
Performance status	ECOG score: 0	1	Reference	—	—
	ECOG score: 1 or 2	0.32	0.07–1.47	—	—
Work status at baseline	Part‐time	1	Reference	—	—
	Full‐time	**3.55**	**1.02–12.31**	—	—

Logistic regression models with the use of backward selection to identify the independent variables. Assumed that a significance level of <0.25 was required for a variable to stay in the model (SLSTAY=0.25).

Included 120 ALaCaRT participants on full‐time / part‐time paid work at baseline.

Abbreviations: ALaCaRT, Australian Laparoscopic Cancer of the Rectum Trial; ECOG, Eastern Cooperative Oncology Group Scale; OR, odds ratio.

### 3.3 Return to preoperative work status or full‐time work

Eighty‐two participants (68%) (laparoscopic *n* = 49; open surgery *n* = 33) returned to their preoperative work status or full‐time work at 12 months. The unadjusted OR for laparoscopic resection was 2.55 (95% CI, 1.15–5.63). However, when adjusted for sociodemographic and clinical factors, the effect of laparoscopic‐assisted surgery was not statistically significant (OR, 2.01; 95% CI, 0.83–4.86). (Table 4).

### 3.4 Sensitivity analysis

In sensitivity analyses, among the 93 participants who returned to work at 12 months, age at randomization (OR, 1.09; 95% CI, 1.01–1.18) was positively associated with return to preoperative work status or full‐time work at 12 months. Both treatment type and surgical outcome had no significant effect on return to preoperative work status or full‐time at 12 months. (Table S1).

Including participants with complete labor force surveys at additional time points of 3 and 6 months (*n* = 102), sensitivity analyses (Table S2) showed participants in paid work at baseline were more likely to RTW at 12 months with laparoscopic‐assisted surgery than open surgery (OR, 6.68; 95% CI, 1.12–39.96). Participants with a BMI of ≥30 kg/m^2^ at baseline compared with those with a BMI of <25 kg/m2 (OR, 24.50; 95% CI, 1.67–358.92) were also more likely to RTW. Those with distant metastases (OR, 0.03; 95% CI, <0.01–0.75) were less likely to RTW during the 12‐month follow‐up period. The effect of a successful resection on RTW was not statistically significant (OR, 5.40; 95% CI, 0.43–68.02). Regression analyses also showed that in this group of rectal cancer survivors who completed surveys at all assessment points, those who received laparoscopic‐assisted surgery (OR, 3.81; 95% CI, 1.09–13.36) compared with those receiving open surgery, and participants who lived with a spouse and dependent children (OR, 18.69; 95% CI, 2.39–145.82) compared with those that lived with their spouse only, were more likely to return to their preoperative work status or full‐time work by 12 months.

Three participants in paid work at baseline died at 12 months and were included in the third sensitivity analysis. Results of logistic regression analysis (Table S3) among 123 participants in paid work at baseline showed those with laparoscopic‐assisted surgery compared with those receiving open surgery were more likely to RTW at 12 months (OR, 3.06; 95% CI, 1.03–9.11). Participants with a successful resection (OR, 4.84; 95% CI, 1.13–20.80) compared with those with an unsuccessful resection and those in full‐time paid work (OR, 4.26; 95% CI, 1.25–14.51) compared with those in part‐time paid work at baseline were more likely to RTW. Those with distant metastases (OR, 0.08; 95% CI, 0.01–0.76) and older participants (OR, 0.95; 95% CI, 0.89–1.00) were less likely to RTW at 12 months. Regression analyses also showed that participants receiving laparoscopic‐assisted surgery (OR, 2.80; 95% CI, 1.07–7.32) compared with open, participants with a successful resection (OR, 5.39; 95% CI, 1.30–22.37) compared with an unsuccessful resection, participants who lived with a spouse and dependent children (OR, 4.44; 95% CI, 1.15–17.13) compared with those that lived with their spouse only, were more likely to return to preoperative work status or full‐time work at 12 months.

## 4 DISCUSSION

Our research identified several factors that were associated with RTW, including being employed in a full‐time rather than part‐time capacity presurgery, and not having distant metastases at time of surgery. As a minimally invasive surgery, faster RTW, which contributes to income, a sense of structure and social recovery,^31^, ^32^, ^33^ has been one of the main objectives of undergoing laparoscopic‐assisted surgery for abdominal and pelvic procedures. Type of surgery, age, sex, and surgical outcome (successful versus unsuccessful), were not significant predictors of RTW for rectal cancer patients in most scenarios. Although the RTW rate was somewhat higher for those who received laparoscopic‐assisted rather than open rectal surgery, this result was not statistically significant in the main analysis, but significant in sensitivity analyses for sub‐groups who completed the labor force survey at all assessment points, and when those who died before 12 months were included.

Findings of this analysis extend our knowledge of laparoscopic surgery for the treatment of rectal cancer. In the context of ALaCaRT results, noninferiority for a surrogate outcome of pathological outcomes was not shown.^19^, ^20^ This analysis showed that laparoscopic‐assisted surgery had no significant improvement on RTW at 12 months, therefore, one might question the ongoing role of laparoscopic‐assisted surgery for the treatment of rectal cancer. However, this approach remains useful for colon cancer,^11^, ^12^, ^13^, ^14^, ^15^, ^16^, ^17^ gastric cancer,^34^ prostate cancer,^35^ and kidney cancer patients.^36^ The survival and recurrence differences in both ALaCaRT and other trials have shown no significant difference between open and laparoscopic approaches.^20^ Given that maintaining employment is an important patient‐centered outcome for cancer survivors, their family members and society,^31^, ^32^, ^33^ the potential impact of laparoscopic‐assisted surgery on RTW could be promoted to new patients.

With some evidence of having less postoperative pain, shorter recovery time and equivalent long‐term outcomes, the laparoscopic approach has become a gold standard for colon cancer,^37^ prostate cancer,^38^ benign ovarian tumors,^39^ endometriosis,^40^ and other surgeries on the organs in the abdominal and pelvic area. For rectal cancer treatment, a Cochrane review based on evidence from nonrandomized studies showed that laparoscopic surgery offered some short‐term benefits in patients with less blood loss, a quicker return to normal diet, less pain, less narcotic use, and less immune response.^41^ In a recent systematic review and meta‐analysis on 13 RCTs, laparoscopic surgery significantly reduced the rate of surgical site infections, blood loss, length of hospital stay and time to first bowel movement, despite it had longer operative time.^42^ This analysis reported the medium‐term impact of such surgical approaches on RTW at 12 months for rectal cancer through a randomized controlled trial.

Novel therapeutic approaches and initiatives such as patient education and group discussion, multidisciplinary intervention through physical exercise and counseling, workplace accommodations through job flexibility, coworker support, and employment‐based health insurance have been implemented to assist cancer survivors to RTW after treatment.^27^, ^32^, ^37^, ^38^, ^39^ This provides a sense of “normality,” a feeling of social belonging,^40^ and relieving financial stress of cancer patients and their family for cancer treatment and other bills, ^43^, ^44^ which can improve cancer survivors’ quality of life.^45^, ^46^ Second, with the rising costs of cancer care,^47^ any policies or practices that improve the likelihood of a person maintaining their employment after cancer treatment deserve thoughtful consideration.

To our knowledge, this is the first evaluative study worldwide using a multicenter randomized controlled trial design to examine the effect of laparoscopic‐assisted surgery on RTW for the treatment of rectal cancer. Importantly, nearly two‐thirds of trial participants were of working age (i.e. 66 years or younger, the current threshold for the Aged Pension in Australia), which is reflective of contemporary issues facing adults with new rectal cancer diagnoses. There are, however, some limitations to acknowledge. First, the study had a modest response rate to the labor force and income impacts of illness questionnaire. Fifty‐one percent of ALaCaRT participants reporting their work status at baseline and 12 months, and some patients had missing data at the 3 and 6 month time points. This was due in part to the late addition of this survey when the trial was already recruiting (after 72 patients). Second, we did not have data about participants’ income protection, where this cover is likely to influence a cancer survivor's decision to RTW. Third, due to the ALaCaRT exclusion criteria, study participants may be healthier than the average rectal cancer survivors in the community; having no T4 tumors, no involvement of the circumferential resection margin, and no concurrent or previous invasive pelvic malignant tumors within 5 years before study enrollment.^19^ Results of this study, therefore, may not be generalizable to all adults treated for rectal cancer. Fourth, the finding of greater RTW for laparoscopic‐assisted surgery may need to be interpreted with caution as there was no evidence this surgical approach had a significant impact on intermediate outcomes (i.e. 2‐year disease‐free survival). ^20^

## 5 CONCLUSIONS

Ongoing employment is a critical concern for many rectal cancer patients and treatment options that enhance RTW prospects should be discussed. Full‐time work and the presence of metastatic disease are likely to predict RTW at 12 months. A laparoscopic‐assisted surgical approach to rectal cancer may facilitate more patients to RTW, however, larger sample sizes are likely needed to confirm this result.

## CONFLICT OF INTEREST

All authors declare no conflict of interest.

## AUTHOR CONTRIBUTIONS

Law: Conceptualization, formal analysis, methodology, software, visualization and writing—original draft; Brewer: Data curation and writing—review and editing; Brown: Data curation, methodology and validation; Wilson: Data curation and project administration; Bailey: Data curation and project administration; Hague: Funding acquisition, investigation, writing—review and editing; Simes: Funding acquisition, investigation, writing—review and editing; Stevenson: Funding acquisition, investigation, writing—review and editing; Solomon: Funding acquisition, investigation, writing—review and editing; Morton: Conceptualization, methodology, supervision, writing—review and editing.

## Supporting information

Table S1‐S3

## Data Availability

Research data are not shared.
