# Supplementary material for: Return to work following laparoscopic‐assisted resection or open resection for rectal cancer: Findings from AlaCaRT—Australasian Laparoscopic Cancer of the Rectum Trial
Source: Cancer Med. 2020 Dec 6;10(2):552–62. doi: 10.1002/cam4.3623 (PMC7877361; doi:10.1002/cam4.3623)
Supplement: Supplementary file 1 — Table S1‐S3 [file CAM4-10-552-s001.docx]

**APPENDICES**

Supplementary Tables

**Table S1: Sensitivity analysis - Multivariable analysis of return to preoperative work status for ALaCaRT participants at 12 months in those that returned to work***

| Variable |  | Return to preoperative work status or full time work at 12 months in those that returned to work (n=93) | |
| --- | --- | --- | --- |
|  |  | OR | 95% CI |
| Treatment arm | Open | 1 | Reference |
|  | Laparoscopic assisted | 1.18 | 0.23 to 6.09 |
| Surgical outcome | Unsuccessful resection | 1 | Reference |
|  | Successful resection | 0.50 | 0.02 to 14.77 |
| Sex | Females | 1 | Reference |
|  | Males | 1.71 | 0.28 to 10.58 |
| Age at randomisation |  | 1.09 | 1.01 to 1.18 |
| Tumour stage | T1 | 1 | Reference |
|  | T2 | 0.00 | 0.00 to 2E180 |
|  | T3 | 0.00 | 0.00 to 1E180 |
| Nodal status | N0 | 1 | Reference |
|  | N1 | 7.32 | 0.59 to 90.85 |
|  | N2 | 1.87 | 0.08 to 61.01 |
| Distant metastasis | M0 | 1 | Reference |
|  | M1 | 24582.55 | 0.000 to I |
| BMI | Below 25 | 1 | Reference |
|  | 25-30 | 0.42 | 0.04 to 4.79 |
|  | >30 | 0.12 | 0.01 to 1.10 |
| Preoperative radiotherapy | No | 1 | Reference |
|  | Yes | **3.25** | **0.51 to 20.96** |
|  |  |  |  |

Abbreviations: OR, odds ratio; BMI, body mass index; ECOG, Eastern Cooperative Oncology Group Scale

*Logistic regression models with the use of hypothesised causal factors and backward selection to identify the independent variables. Assumed that a significance level of p<0.25 required for a variable to stay in the model (SLSTAY=0.25).

b Included 93 ALaCaRT participants who worked full-time/ part-time at baseline and returned to work at 12 months

**Table S2: Sensitivity analysis - Multivariable analysis of return to work and return to preoperative work status for ALaCaRT participants at 12 months for participants with complete return to work data including at all time points (baseline, 3 months, 6 months and 12 months)***

| Variable |  | Return to work at 12 months (n=102) | | Return to preoperative work status or full-time work at 12 months (n=102) | |
| --- | --- | --- | --- | --- | --- |
|  |  | OR | 95% CI | OR | 95%CI |
| Treatment arm | Open | 1 | Reference | 1 | Reference |
|  | Laparoscopic assisted | **6.68** | **1.12 to 39.96** | **3.81** | **1.09 to 13.36** |
| Surgical outcome | Unsuccessful resection | 1 | Reference | 1 | Reference |
|  | Successful resection | 5.40 | 0.43 to 68.02 | 5.95 | 0.85 to 41.72 |
| Sex | Females | 1 | Reference | 1 | Reference |
|  | Males | 2.52 | 0.40 to 15.75 | 2.43 | 0.67 to 8.81 |
| Age at randomization (years) |  | 0.94 | 0.87 to 1.02 | 0.80 | 0.05 to 12.58 |
| Tumour stage | T1 | 1 | Reference | 1 | Reference |
|  | T2 | 1.53 | 0.08 to 27.91 | 0.80 | 0.01 to 1.95 |
|  | T3 | 0.21 | 0.01 to 3.80 | 0.11 | 0.38 to 8.03 |
| Nodal status | N0 | 1 | Reference | 1 | Reference |
|  | N1 | 0.60 | 0.08 to 4.48 | 1.74 | 0.29 to 5.56 |
|  | N2 | 17.28 | 0.83 to 359.64 | 6.42 | 0.68 to 60.78 |
| Distant metastasis | M0 | 1 | Reference | 1 | Reference |
|  | M1 | **0.03** | **<0.001 to 0.75** | 0.35 | 0.02 to 5.60 |
| BMI | Below 25 | 1 | Reference | 1 | Reference |
|  | 25-30 | 2.01 | 0.34 to 11.82 | 1.84 | 0.44 to 7.63 |
|  | >30 | **24.50** | **1.67 to 358.92** | 4.24 | 0.85 to 21.21 |
| Preoperative radiotherapy | No | - | - | 1 | Reference |
|  | Yes | - | - | 2.82 | 0.68 to 11.79 |
| Performance status | ECOG score: 0 | 1 | Reference | 1 | Reference |
|  | ECOG score: 1 or 2 | 0.18 | 0.02 to 1.55 | 0.21 | 0.02 to 2.57 |
| Work status at baseline | Part-time | 1 | Reference |  |  |
|  | Full-time | 4.03 | 0.63 to 25.82 |  |  |
| Education | Year 9 or below / never attended school | 1 | Reference | 1 | Reference |
|  | High school | 2.51 | 0.13 to 48.78 | 1.05 | 0.10 to 10.87 |
|  | Certificate / Diploma | 0.20 | 0.01 to 3.54 | 0.21 | 0.02 to 2.57 |
|  | University Degree | 1.55 | 0.06 to 37.04 | 0.19 | 0.02 to 2.35 |
| Family composition | Couple only | - | - | 1 | Reference |
|  | Couple with dependent children | - | - | **18.69** | **2.39 to 145.82** |
|  | Single parent | - | - | 5.21 | 0.26 to 105.84 |
|  | Living alone | - | - | 1.64 | 0.30 to 9.09 |

Abbreviations: OR, odds ratio; BMI, body mass index; ECOG, Eastern Cooperative Oncology Group Scale

*Logistic regression models with the use of hypothesised causal factors and backward selection to identify independent variables. Assumed that a significance level of p<0.25 was required for a variable to stay in the model (SLSTAY=0.25)

Included 102 ALaCaRT participants in full-time or part-time paid work at baseline

**Table S3: Sensitivity analysis - Multivariable analysis of return to work and return to preoperative work status for ALaCaRT participants at 12 months (including those who died by 12 months)***

| Variable |  | Return to work at 12 months  (n=123) | | Return to preoperative work status or full-time work at 12 months (n=123) | |
| --- | --- | --- | --- | --- | --- |
|  |  | OR | 95% CI | OR | 95% CI |
| Treatment arm | Open | 1 | Reference | 1 | Reference |
|  | Laparoscopic assisted | **3.06** | **1.03 to 9.11** | **2.80** | **1.07 to 7.32** |
| Surgical outcome | Unsuccessful resection | 1 | Reference | 1 | Reference |
|  | Successful resection | **4.84** | **1.13 to 20.80** | **5.39** | **1.30 to 22.37** |
| Sex | Females | 1 | Reference | 1 | Reference |
|  | Males | 1.06 | 0.31 to 3.63 | 1.21 | 0.42 to 3.47 |
| Age (yrs) at randomisation |  | **0.95** | **0.89 to 1.00** | 0.99 | 0.94 to 1.04 |
| Tumour stage | T1 | 1 | Reference | 1 | Reference |
|  | T2 | 2.26 | 0.36 to 18.32 | 1.77 | 0.26 to 12.01 |
|  | T3 | 1.21 | 0.17 to 8.51 | 0.50 | 0.07 to 3.33 |
| Nodal status | N0 | 1 | Reference | 1 | Reference |
|  | N1 | 0.58 | 0.15 to 2.20 | 1.40 | 0.43 to 4.57 |
|  | N2 | 6.24 | 0.70 to 55.44 | 5.72 | 0.97 to 33.75 |
| Distant metastasis | M0 | 1 | Reference | 1 | Reference |
|  | M1 | **0.08** | **0.01 to 0.76** | 0.28 | 0.04 to 2.20 |
| Performance status | ECOG score: 0 | 1 | Reference | - | - |
|  | ECOG score: 1 or 2 | 0.35 | 0.08 to 1.59 | - | - |
| Work status at baseline | Part-time | 1 | Reference | 1 | Reference |
|  | Full-time | **4.26** | **1.25 to 14.51** | 2.07 | 0.67 to 6.42 |
| Education | Year 9 or below / never attended school | - | - | 1 | Reference |
|  | High school | - | - | 0.62 | 0.09 to 4.40 |
|  | Certificate / Diploma | - | - | 0.26 | 0.03 to 2.14 |
|  | University Degree | - | - | 0.20 | 0.02 to 1.63 |
| Family composition | Couple only | - | - | 1 | Reference |
|  | Couple with dependent children | - | - | **4.44** | **1.15 to 17.13** |
|  | Single parent | - | - | 1.28 | 0.09 to 17.92 |
|  | Living alone | - | - | 1.43 | 0.42 to 4.90 |
|  |  |  |  |  |  |

Abbreviations: OR, odds ratio; ECOG, Eastern Cooperative Oncology Group Scale

*Logistic regression models with the use of backward selection to identify the independent variables. Assumed that a significance level of <0.25 was required for a variable to stay in the model (SLSTAY=0.25).

Included 123 ALaCaRT participants on full-time / part-time paid work at baseline; 3 of these participants died during the 12-month follow-up period and their return-to-work/ return-to-preoperative work status were defined as negative in this analysis.
